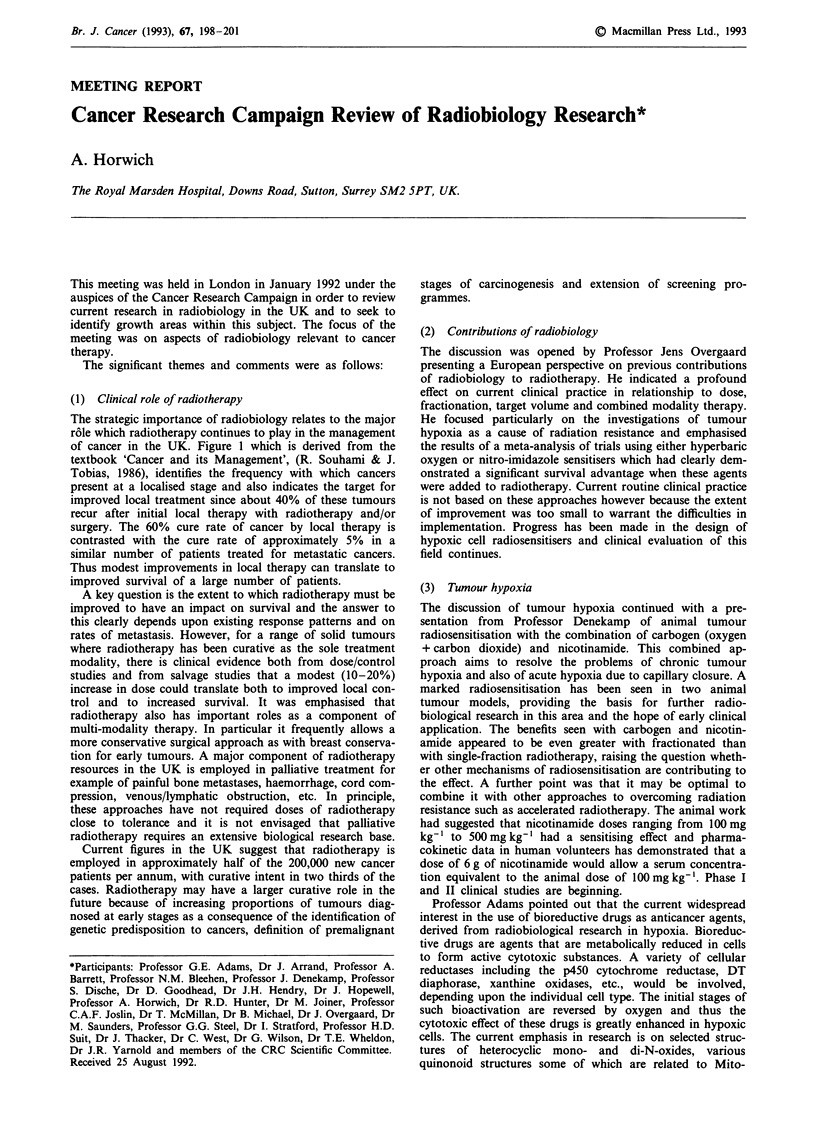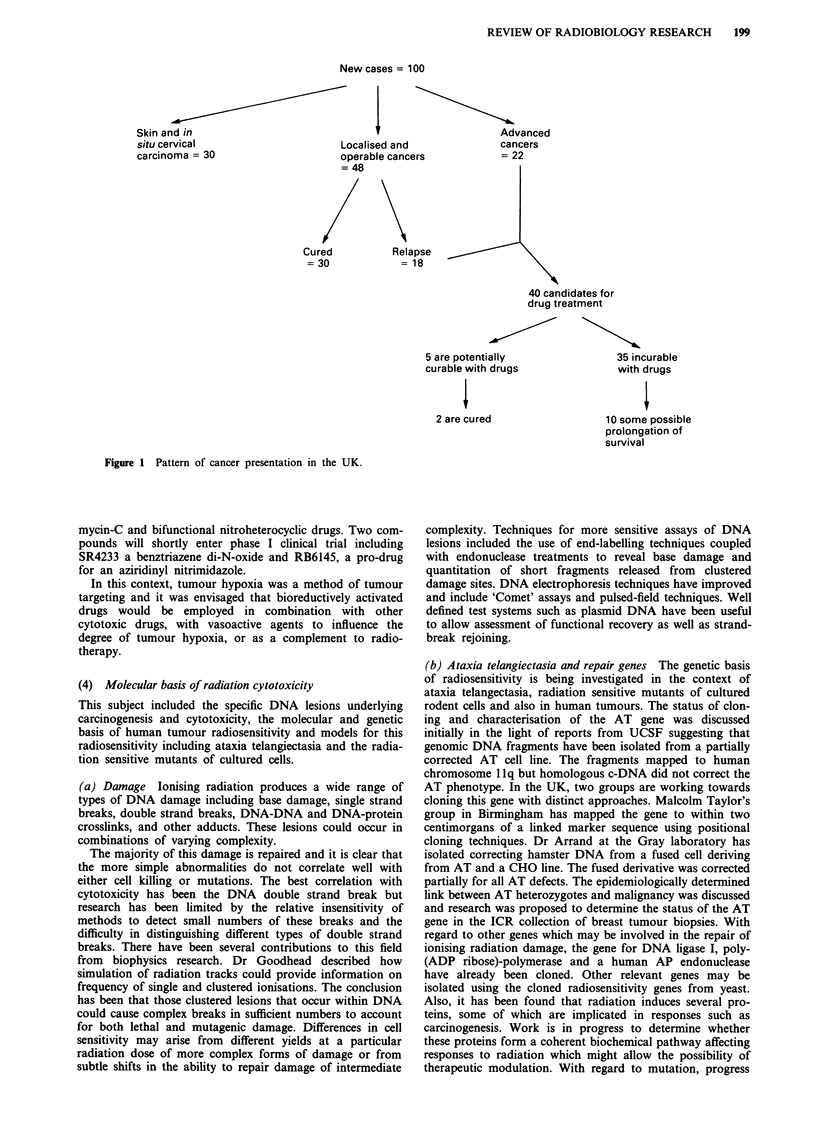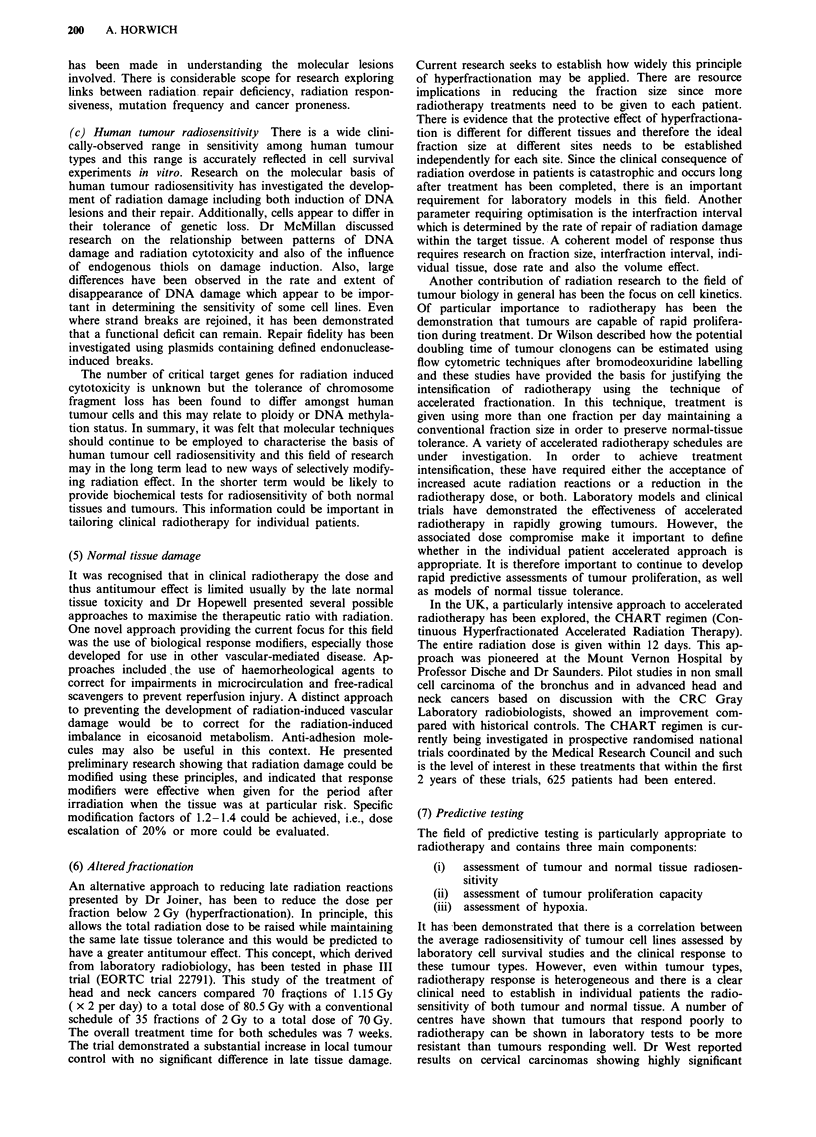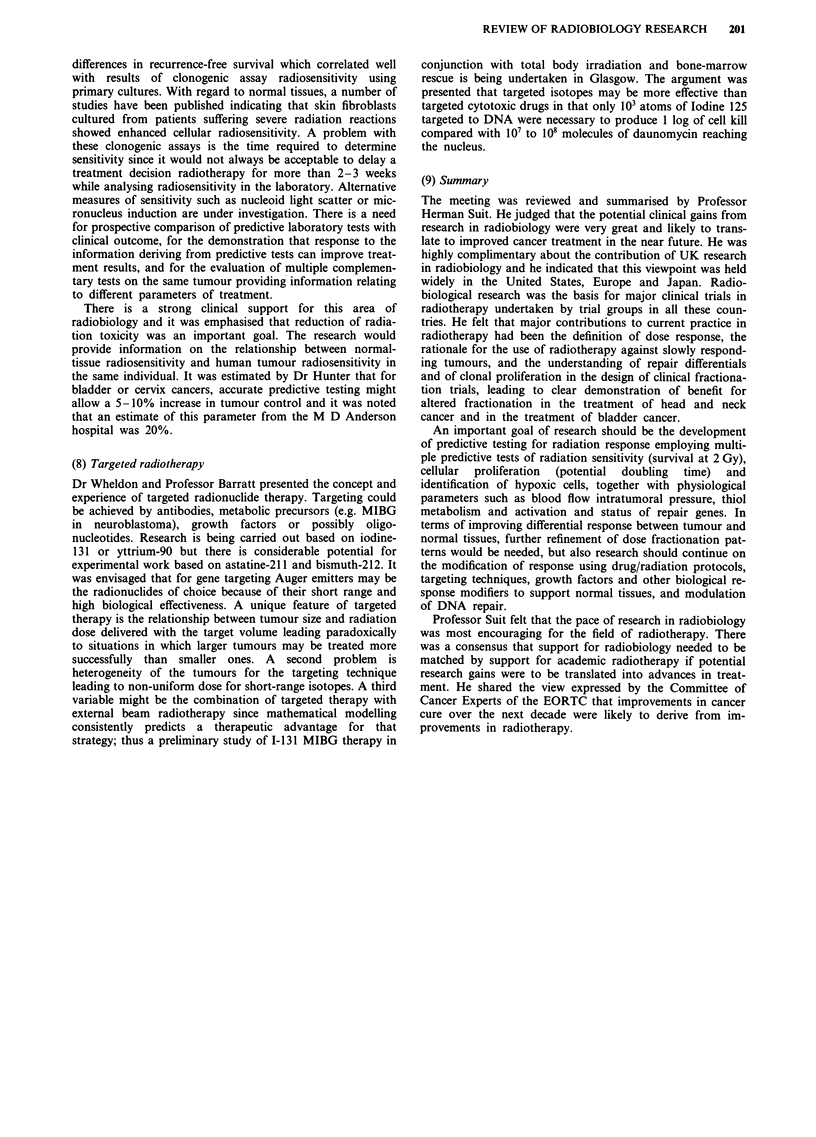# Cancer Research Campaign review of radiobiology research.

**DOI:** 10.1038/bjc.1993.34

**Published:** 1993-01

**Authors:** A. Horwich

**Affiliations:** Royal Marsden Hospital, Sutton, Surrey, UK.

## Abstract

The meeting was reviewed and summarised by Professor Herman Suit. He judged that the potential clinical gains from research in radiobiology were very great and likely to translate to improved cancer treatment in the near future. He was highly complimentary about the contribution of UK research in radiobiology and he indicated that this viewpoint was held widely in the United States, Europe and Japan. Radiobiological research was the basis for major clinical trials in radiotherapy undertaken by trial groups in all these countries. He felt that major contributions to current practice in radiotherapy had been the definition of dose response, the rationale for the use of radiotherapy against slowly responding tumours, and the understanding of repair differentials and of clonal proliferation in the design of clinical fractionation trials, leading to clear demonstration of benefit for altered fractionation in the treatment of head and neck cancer and in the treatment of bladder cancer. An important goal of research should be the development of predictive testing for radiation response employing multiple predictive tests of radiation sensitivity (survival at 2 Gy), cellular proliferation (potential doubling time) and identification of hypoxic cells, together with physiological parameters such as blood flow intratumoral pressure, thiol metabolism and activation and status of repair genes. In terms of improving differential response between tumour and normal tissues, further refinement of dose fractionation patterns would be needed, but also research should continue on the modification of response using drug/radiation protocols, targeting techniques, growth factors and other biological response modifiers to support normal tissues, and modulation of DNA repair. Professor Suit felt that the pace of research in radiobiology was most encouraging for the field of radiotherapy. There was a consensus that support for radiobiology needed to be matched by support for academic radiotherapy if potential research gains were to be translated into advances in treatment. He shared the view expressed by the Committee of Cancer Experts of the EORTC that improvements in cancer cure over the next decade were likely to derive from improvements in radiotherapy.


MEETING REPORT

### Cancer Research Campaign Review of Radiobiology Research*

A. Horwich

*The Royal Marsden Hospital, Downs Road, Sutton, Surrey SM2 5PT, UK.*

This meeting was held in London in January 1992 under the auspices of the Cancer Research Campaign in order to review current research in radiobiology in the UK and to seek to identify growth areas within this subject. The focus of the meeting was on aspects of radiobiology relevant to cancer therapy.

The significant themes and comments were as follows:

### (1) *Clinical role of radiotherapy*

The strategic importance of radiobiology relates to the major rôle which radiotherapy continues to play in the management of cancer in the UK. Figure 1 which is derived from the textbook 'Cancer and its Management', (R. Souhami & J. Tobias, 1986), identifies the frequency with which cancers present at a localised stage and also indicates the target for improved local treatment since about 40% of these tumours recur after initial local therapy with radiotherapy and/or surgery. The 60% cure rate of cancer by local therapy is contrasted with the cure rate of approximately 5% in a similar number of patients treated for metastatic cancers. Thus modest improvements in local therapy can translate to improved survival of a large number of patients.

A key question is the extent to which radiotherapy must be improved to have an impact on survival and the answer to this clearly depends upon existing response patterns and on rates of metastasis. However, for a range of solid tumours where radiotherapy has been curative as the sole treatment modality, there is clinical evidence both from dose/control studies and from salvage studies that a modest (10-20%) increase in dose could translate both to improved local control and to increased survival. It was emphasised that radiotherapy also has important roles as a component of multi-modality therapy. In particular it frequently allows a more conservative surgical approach as with breast conservation for early tumours. A major component of radiotherapy resources in the UK is employed in palliative treatment for example of painful bone metastases, haemorrhage, cord compression, venous/lymphatic obstruction, etc. In principle, these approaches have not required doses of radiotherapy close to tolerance and it is not envisaged that palliative radiotherapy requires an extensive biological research base.

Current figures in the UK suggest that radiotherapy is employed in approximately half of the 200,000 new cancer patients per annum, with curative intent in two thirds of the cases. Radiotherapy may have a larger curative role in the future because of increasing proportions of tumours diagnosed at early stages as a consequence of the identification of genetic predisposition to cancers, definition of premalignant stages of carcinogenesis and extension of screening programmes.

### (2) *Contributions of radiobiology*

The discussion was opened by Professor Jens Overgaard presenting a European perspective on previous contributions of radiobiology to radiotherapy. He indicated a profound effect on current clinical practice in relationship to dose, fractionation, target volume and combined modality therapy. He focused particularly on the investigations of tumour hypoxia as a cause of radiation resistance and emphasised the results of a meta-analysis of trials using either hyperbaric oxygen or nitro-imidazole sensitisers which had clearly demonstrated a significant survival advantage when these agents were added to radiotherapy. Current routine clinical practice is not based on these approaches however because the extent of improvement was too small to warrant the difficulties in implementation. Progress has been made in the design of hypoxic cell radiosensitisers and clinical evaluation of this field continues.

### (3) *Tumour hypoxia*

The discussion of tumour hypoxia continued with a presentation from Professor Denekamp of animal tumour radiosensitisation with the combination of carbogen (oxygen + carbon dioxide) and nicotinamide. This combined approach aims to resolve the problems of chronic tumour hypoxia and also of acute hypoxia due to capillary closure. A marked radiosensitisation has been seen in two animal tumour models, providing the basis for further radiobiological research in this area and the hope of early clinical application. The benefits seen with carbogen and nicotinamide appeared to be even greater with fractionated than with single-fraction radiotherapy, raising the question whether other mechanisms of radiosensitisation are contributing to the effect. A further point was that it may be optimal to combine it with other approaches to overcoming radiation resistance such as accelerated radiotherapy. The animal work had suggested that nicotinamide doses ranging from 100 mg $kg^{-1}$ to 500 mg $kg^{-1}$ had a sensitising effect and pharmacokinetic data in human volunteers has demonstrated that a dose of 6 g of nicotinamide would allow a serum concentration equivalent to the animal dose of 100 mg $kg^{-1}$. Phase I and II clinical studies are beginning.

Professor Adams pointed out that the current widespread interest in the use of bioreductive drugs as anticancer agents, derived from radiobiological research in hypoxia. Bioreductive drugs are agents that are metabolically reduced in cells to form active cytotoxic substances. A variety of cellular reductases including the p450 cytochrome reductase, DT diaphorase, xanthine oxidases, etc., would be involved, depending upon the individual cell type. The initial stages of such bioactivation are reversed by oxygen and thus the cytotoxic effect of these drugs is greatly enhanced in hypoxic cells. The current emphasis in research is on selected structures of heterocyclic mono- and di-N-oxides, various quinonoid structures some of which are related to Mito-

*Participants: Professor G.E. Adams, Dr J. Arrand, Professor A. Barrett, Professor N.M. Bleehen, Professor J. Denekamp, Professor S. Dische, Dr D. Goodhead, Dr J.H. Hendry, Dr J. Hopewell, Professor A. Horwich, Dr R.D. Hunter, Dr M. Joiner, Professor C.A.F. Joslin, Dr T. McMillan, Dr B. Michael, Dr J. Overgaard, Dr M. Saunders, Professor G.G. Steel, Dr I. Stratford, Professor H.D. Suit, Dr J. Thacker, Dr C. West, Dr G. Wilson, Dr T.E. Wheldon, Dr J.R. Yarnold and members of the CRC Scientific Committee.
Received 25 August 1992.

**Figure 1** Pattern of cancer presentation in the UK.

mycin-C and bifunctional nitroheterocyclic drugs. Two compounds will shortly enter phase I clinical trial including SR4233 a benztriazene di-N-oxide and RB6145, a pro-drug for an aziridinyl nitrimidazole.

In this context, tumour hypoxia was a method of tumour targeting and it was envisaged that bioreductively activated drugs would be employed in combination with other cytotoxic drugs, with vasoactive agents to influence the degree of tumour hypoxia, or as a complement to radiotherapy.

### (4) *Molecular basis of radiation cytotoxicity*

This subject included the specific DNA lesions underlying carcinogenesis and cytotoxicity, the molecular and genetic basis of human tumour radiosensitivity and models for this radiosensitivity including ataxia telangiectasia and the radiation sensitive mutants of cultured cells.

*(a) Damage* Ionising radiation produces a wide range of types of DNA damage including base damage, single strand breaks, double strand breaks, DNA-DNA and DNA-protein crosslinks, and other adducts. These lesions could occur in combinations of varying complexity.

The majority of this damage is repaired and it is clear that the more simple abnormalities do not correlate well with either cell killing or mutations. The best correlation with cytotoxicity has been the DNA double strand break but research has been limited by the relative insensitivity of methods to detect small numbers of these breaks and the difficulty in distinguishing different types of double strand breaks. There have been several contributions to this field from biophysics research. Dr Goodhead described how simulation of radiation tracks could provide information on frequency of single and clustered ionisations. The conclusion has been that those clustered lesions that occur within DNA could cause complex breaks in sufficient numbers to account for both lethal and mutagenic damage. Differences in cell sensitivity may arise from different yields at a particular radiation dose of more complex forms of damage or from subtle shifts in the ability to repair damage of intermediate complexity. Techniques for more sensitive assays of DNA lesions included the use of end-labelling techniques coupled with endonuclease treatments to reveal base damage and quantitation of short fragments released from clustered damage sites. DNA electrophoresis techniques have improved and include 'Comet' assays and pulsed-field techniques. Well defined test systems such as plasmid DNA have been useful to allow assessment of functional recovery as well as strand-break rejoining.

*(b) Ataxia telangiectasia and repair genes* The genetic basis of radiosensitivity is being investigated in the context of ataxia telangectasia, radiation sensitive mutants of cultured rodent cells and also in human tumours. The status of cloning and characterisation of the AT gene was discussed initially in the light of reports from UCSF suggesting that genomic DNA fragments have been isolated from a partially corrected AT cell line. The fragments mapped to human chromosome 11q but homologous c-DNA did not correct the AT phenotype. In the UK, two groups are working towards cloning this gene with distinct approaches. Malcolm Taylor's group in Birmingham has mapped the gene to within two centimorgans of a linked marker sequence using positional cloning techniques. Dr Arrand at the Gray laboratory has isolated correcting hamster DNA from a fused cell deriving from AT and a CHO line. The fused derivative was corrected partially for all AT defects. The epidemiologically determined link between AT heterozygotes and malignancy was discussed and research was proposed to determine the status of the AT gene in the ICR collection of breast tumour biopsies. With regard to other genes which may be involved in the repair of ionising radiation damage, the gene for DNA ligase I, poly-(ADP ribose)-polymerase and a human AP endonuclease have already been cloned. Other relevant genes may be isolated using the cloned radiosensitivity genes from yeast. Also, it has been found that radiation induces several proteins, some of which are implicated in responses such as carcinogenesis. Work is in progress to determine whether these proteins form a coherent biochemical pathway affecting responses to radiation which might allow the possibility of therapeutic modulation. With regard to mutation, progress has been made in understanding the molecular lesions involved. There is considerable scope for research exploring links between radiation repair deficiency, radiation responsiveness, mutation frequency and cancer proneness.

*(c) Human tumour radiosensitivity* There is a wide clinically-observed range in sensitivity among human tumour types and this range is accurately reflected in cell survival experiments *in vitro*. Research on the molecular basis of human tumour radiosensitivity has investigated the development of radiation damage including both induction of DNA lesions and their repair. Additionally, cells appear to differ in their tolerance of genetic loss. Dr McMillan discussed research on the relationship between patterns of DNA damage and radiation cytotoxicity and also of the influence of endogenous thiols on damage induction. Also, large differences have been observed in the rate and extent of disappearance of DNA damage which appear to be important in determining the sensitivity of some cell lines. Even where strand breaks are rejoined, it has been demonstrated that a functional deficit can remain. Repair fidelity has been investigated using plasmids containing defined endonuclease-induced breaks.

The number of critical target genes for radiation induced cytotoxicity is unknown but the tolerance of chromosome fragment loss has been found to differ amongst human tumour cells and this may relate to ploidy or DNA methylation status. In summary, it was felt that molecular techniques should continue to be employed to characterise the basis of human tumour cell radiosensitivity and this field of research may in the long term lead to new ways of selectively modifying radiation effect. In the shorter term would be likely to provide biochemical tests for radiosensitivity of both normal tissues and tumours. This information could be important in tailoring clinical radiotherapy for individual patients.

### (5) *Normal tissue damage*

It was recognised that in clinical radiotherapy the dose and thus antitumour effect is limited usually by the late normal tissue toxicity and Dr Hopewell presented several possible approaches to maximise the therapeutic ratio with radiation. One novel approach providing the current focus for this field was the use of biological response modifiers, especially those developed for use in other vascular-mediated disease. Approaches included the use of haemorheological agents to correct for impairments in microcirculation and free-radical scavengers to prevent reperfusion injury. A distinct approach to preventing the development of radiation-induced vascular damage would be to correct for the radiation-induced imbalance in eicosanoid metabolism. Anti-adhesion molecules may also be useful in this context. He presented preliminary research showing that radiation damage could be modified using these principles, and indicated that response modifiers were effective when given for the period after irradiation when the tissue was at particular risk. Specific modification factors of 1.2–1.4 could be achieved, i.e., dose escalation of 20% or more could be evaluated.

### (6) *Altered fractionation*

An alternative approach to reducing late radiation reactions presented by Dr Joiner, has been to reduce the dose per fraction below 2 Gy (hyperfractionation). In principle, this allows the total radiation dose to be raised while maintaining the same late tissue tolerance and this would be predicted to have a greater antitumour effect. This concept, which derived from laboratory radiobiology, has been tested in phase III trial (EORTC trial 22791). This study of the treatment of head and neck cancers compared 70 fractions of 1.15 Gy ($\times$ 2 per day) to a total dose of 80.5 Gy with a conventional schedule of 35 fractions of 2 Gy to a total dose of 70 Gy. The overall treatment time for both schedules was 7 weeks. The trial demonstrated a substantial increase in local tumour control with no significant difference in late tissue damage. Current research seeks to establish how widely this principle of hyperfractionation may be applied. There are resource implications in reducing the fraction size since more radiotherapy treatments need to be given to each patient. There is evidence that the protective effect of hyperfractionation is different for different tissues and therefore the ideal fraction size at different sites needs to be established independently for each site. Since the clinical consequence of radiation overdose in patients is catastrophic and occurs long after treatment has been completed, there is an important requirement for laboratory models in this field. Another parameter requiring optimisation is the interfraction interval which is determined by the rate of repair of radiation damage within the target tissue. A coherent model of response thus requires research on fraction size, interfraction interval, individual tissue, dose rate and also the volume effect.

Another contribution of radiation research to the field of tumour biology in general has been the focus on cell kinetics. Of particular importance to radiotherapy has been the demonstration that tumours are capable of rapid proliferation during treatment. Dr Wilson described how the potential doubling time of tumour clonogens can be estimated using flow cytometric techniques after bromodeoxuridine labelling and these studies have provided the basis for justifying the intensification of radiotherapy using the technique of accelerated fractionation. In this technique, treatment is given using more than one fraction per day maintaining a conventional fraction size in order to preserve normal-tissue tolerance. A variety of accelerated radiotherapy schedules are under investigation. In order to achieve treatment intensification, these have required either the acceptance of increased acute radiation reactions or a reduction in the radiotherapy dose, or both. Laboratory models and clinical trials have demonstrated the effectiveness of accelerated radiotherapy in rapidly growing tumours. However, the associated dose compromise make it important to define whether in the individual patient accelerated approach is appropriate. It is therefore important to continue to develop rapid predictive assessments of tumour proliferation, as well as models of normal tissue tolerance.

In the UK, a particularly intensive approach to accelerated radiotherapy has been explored, the CHART regimen (Continuous Hyperfractionated Accelerated Radiation Therapy). The entire radiation dose is given within 12 days. This approach was pioneered at the Mount Vernon Hospital by Professor Dische and Dr Saunders. Pilot studies in non small cell carcinoma of the bronchus and in advanced head and neck cancers based on discussion with the CRC Gray Laboratory radiobiologists, showed an improvement compared with historical controls. The CHART regimen is currently being investigated in prospective randomised national trials coordinated by the Medical Research Council and such is the level of interest in these treatments that within the first 2 years of these trials, 625 patients had been entered.

### (7) *Predictive testing*

The field of predictive testing is particularly appropriate to radiotherapy and contains three main components:

(i) assessment of tumour and normal tissue radiosensitivity
(ii) assessment of tumour proliferation capacity
(iii) assessment of hypoxia.

It has been demonstrated that there is a correlation between the average radiosensitivity of tumour cell lines assessed by laboratory cell survival studies and the clinical response to these tumour types. However, even within tumour types, radiotherapy response is heterogeneous and there is a clear clinical need to establish in individual patients the radiosensitivity of both tumour and normal tissue. A number of centres have shown that tumours that respond poorly to radiotherapy can be shown in laboratory tests to be more resistant than tumours responding well. Dr West reported results on cervical carcinomas showing highly significant differences in recurrence-free survival which correlated well with results of clonogenic assay radiosensitivity using primary cultures. With regard to normal tissues, a number of studies have been published indicating that skin fibroblasts cultured from patients suffering severe radiation reactions showed enhanced cellular radiosensitivity. A problem with these clonogenic assays is the time required to determine sensitivity since it would not always be acceptable to delay a treatment decision radiotherapy for more than 2–3 weeks while analysing radiosensitivity in the laboratory. Alternative measures of sensitivity such as nucleoid light scatter or micronucleus induction are under investigation. There is a need for prospective comparison of predictive laboratory tests with clinical outcome, for the demonstration that response to the information deriving from predictive tests can improve treatment results, and for the evaluation of multiple complementary tests on the same tumour providing information relating to different parameters of treatment.

There is a strong clinical support for this area of radiobiology and it was emphasised that reduction of radiation toxicity was an important goal. The research would provide information on the relationship between normal-tissue radiosensitivity and human tumour radiosensitivity in the same individual. It was estimated by Dr Hunter that for bladder or cervix cancers, accurate predictive testing might allow a 5–10% increase in tumour control and it was noted that an estimate of this parameter from the M D Anderson hospital was 20%.

(8) *Targeted radiotherapy*

Dr Wheldon and Professor Barratt presented the concept and experience of targeted radionuclide therapy. Targeting could be achieved by antibodies, metabolic precursors (e.g. MIBG in neuroblastoma), growth factors or possibly oligonucleotides. Research is being carried out based on iodine-131 or yttrium-90 but there is considerable potential for experimental work based on astatine-211 and bismuth-212. It was envisaged that for gene targeting Auger emitters may be the radionuclides of choice because of their short range and high biological effectiveness. A unique feature of targeted therapy is the relationship between tumour size and radiation dose delivered with the target volume leading paradoxically to situations in which larger tumours may be treated more successfully than smaller ones. A second problem is heterogeneity of the tumours for the targeting technique leading to non-uniform dose for short-range isotopes. A third variable might be the combination of targeted therapy with external beam radiotherapy since mathematical modelling consistently predicts a therapeutic advantage for that strategy; thus a preliminary study of I-131 MIBG therapy in conjunction with total body irradiation and bone-marrow rescue is being undertaken in Glasgow. The argument was presented that targeted isotopes may be more effective than targeted cytotoxic drugs in that only $10^3$ atoms of Iodine 125 targeted to DNA were necessary to produce 1 log of cell kill compared with $10^7$ to $10^8$ molecules of daunomycin reaching the nucleus.

(9) *Summary*

The meeting was reviewed and summarised by Professor Herman Suit. He judged that the potential clinical gains from research in radiobiology were very great and likely to translate to improved cancer treatment in the near future. He was highly complimentary about the contribution of UK research in radiobiology and he indicated that this viewpoint was held widely in the United States, Europe and Japan. Radiobiological research was the basis for major clinical trials in radiotherapy undertaken by trial groups in all these countries. He felt that major contributions to current practice in radiotherapy had been the definition of dose response, the rationale for the use of radiotherapy against slowly responding tumours, and the understanding of repair differentials and of clonal proliferation in the design of clinical fractionation trials, leading to clear demonstration of benefit for altered fractionation in the treatment of head and neck cancer and in the treatment of bladder cancer.

An important goal of research should be the development of predictive testing for radiation response employing multiple predictive tests of radiation sensitivity (survival at 2 Gy), cellular proliferation (potential doubling time) and identification of hypoxic cells, together with physiological parameters such as blood flow intratumoral pressure, thiol metabolism and activation and status of repair genes. In terms of improving differential response between tumour and normal tissues, further refinement of dose fractionation patterns would be needed, but also research should continue on the modification of response using drug/radiation protocols, targeting techniques, growth factors and other biological response modifiers to support normal tissues, and modulation of DNA repair.

Professor Suit felt that the pace of research in radiobiology was most encouraging for the field of radiotherapy. There was a consensus that support for radiobiology needed to be matched by support for academic radiotherapy if potential research gains were to be translated into advances in treatment. He shared the view expressed by the Committee of Cancer Experts of the EORTC that improvements in cancer cure over the next decade were likely to derive from improvements in radiotherapy.